# 胸腔镜辅助小切口治疗多形性肺癌28例疗效观察

**DOI:** 10.3779/j.issn.1009-3419.2010.08.12

**Published:** 2010-08-20

**Authors:** 向阳 成, 信国 熊, 源 邱, 君 刘, 汉章 陈, 建行 何

**Affiliations:** 510120 广州，广州医学院第一附属医院胸外科，广州呼吸疾病研究所 Department of Cardiothoracic Surgery, the First Afliated Hospital of Guangzhou Medical College, Guangzhou Institute of Respiratory Disease & China State Key Laboratory of Respiratory Disease, Guangzhou 510120, China

**Keywords:** 胸腔镜辅助小切口手术, 多形性肺癌, 根治术, Video-assisted mini-thoracoscopy, Pleomorphic carcinoma of the lung, Radical resection

## Abstract

**背景与目的:**

胸腔镜辅助小切口（video-assisted mini-thoracoscopy, VAMT）下完成肺叶切除已有二十年的历史，具有创伤小、术后疼痛轻、肺功能损失少、恢复快等优点，是目前微创胸部外科的发展趋势。本研究旨在探讨胸腔镜辅助小切口肺叶切除术在多形性肺癌治疗中的应用价值。

**方法:**

应用VAMT行根治性肺叶切除术治疗28例多形性肺癌。采用常规器械与胸腔镜器械相结合直视操作处理肺血管、支气管行肺叶切除，胸腔镜、直视下行纵隔、肺门区淋巴结清扫。

**结果:**

28例均获成功。无围手术期死亡，术中出血量200 mL-450 mL，平均300 mL；胸管留置时间3 d-8 d，平均5 d，术后总引流量300 mL-800 mL，平均460 mL；术后住院7 d-14 d，平均12 d。随访28例，其中2例患者于术后2个月死亡，其余患者生存均超过1年，3年生存率为60.7%（95%CI: 40.3%-81.1%）。

**结论:**

VAMT治疗多形性肺癌近期疗效良好，既发挥了微创外科的优越性，又达到了传统开胸手术安全、可靠的效果，清扫淋巴结符合肿瘤手术原则，有很好的应用前景。

## Abstract (en)

**Background and objective:**

Video-assisted mini-thoracoscopy (VAMT) has been used for pulmonary lobectomy for 20 years, which has many merits including small wound, less pain and quick recovery. VAMT is the tendency of pectoral minimally invasive surgical treatment. The aim of this study is to evaluate the efficacy of video-assisted mini-thoracoscopy (VAMT) for radical resection of pleomorphic carcinoma of the lung.

**Methods:**

Complete resection of pleomorphic carcinoma of the lung was performed on 28 patients by VMAT. A 1.5 cm and a 6 cm to 8 cm incision was made during the operation. Both standard surgical instruments and thoracoscopic set were used to treat the pulmonary vessels, perform lobotomy, and remove the lymph nodes in the mediastinum and pulmonary portal.

**Results:**

The operation was completed in all of the cases. No peri-operative death occurred. The total volume of hemorrhage was 200 mL to 450 mL (mean, 300 mL). The patients received chest drainage for 3 to 8 days after the operation (mean, 5 d). The time in hospital was 7 d-14 d (mean, 12 d). Five-year overall survival and disease-free survival were 39.2% and 47.1%, respectively. Follow-up was available in all 28 patients for up to 2 to 91 months. Among the 28 cases, only 2 patients died 2 months after the operation, the rest all live up over 1 year. 3-year survival rate was 60.7% (95%CI: 40.3%-81.1%).

**Conclusion:**

VAMT is effective for radical resection of pleomorphic carcinoma of the lung in a short term. Combination the superiority of traditional procedure and VATS, so VAMT is safe and reliable for radical resection of pleomorphic carcinoma of the lung.

肺多形性癌是一种分化差的含有肉瘤样成分的非小细胞癌，其本质是癌，较少见，约占原发性肺癌的1%以下^[1, 2]^。平均发病年龄为60岁，以男性为主。组织学上显示恶性上皮性和同源性肉瘤样梭形和/或巨细胞成分，如低分化鳞状细胞癌、腺癌或大细胞癌中含有梭形或巨细胞成分，或者是仅为梭形或巨细胞组成的癌。此类患者预后较差，近年来随着电视胸腔镜手术（video-assisted thoracic surgery, VATS）的发展（VATS具有创伤小、出血、输血少、恢复快且符合美容原则等特点），显著提高了肺癌的诊治水平，以及胸外科医生技术的日益熟练，实施更为复杂的肺切除术将成为可能。电视胸腔镜辅助小切口手术（video-assisted mini-thoracoscopy, VAMT）结合了VATS和传统开胸手术的优点。2000年6月-2007年12月，我院执行VAMT进行解剖学肺叶切除联合淋巴结清扫治疗28例多形性肺癌患者，取得了良好的近期疗效，现报道如下。

## 1 资料与方法

### 1.1 一般资料

本组肺多形性癌28例（术前有明确的病理诊断或术中快速证实方可纳入本组资料），均为本院于2000年6月-2007年12月收治的病例，其中男性19例，女性9例，年龄38岁-84岁，平均58.1岁。所有患者按胸腔镜和开胸手术准备，先行胸腔镜探查，未明确诊断者胸腔镜活检，行冰冻病理检查。按照肺癌的国际TNM分期标准（2009年第7版）：Ⅰa期4例，Ⅰb期9例，Ⅱa期4例，Ⅱb期5例，Ⅲa期5例，Ⅳ期1例。肿瘤位于右肺上叶6例，右肺中叶2例，右肺下叶6例，左肺上叶9例，左肺下叶5例。术前均常规经胸部CT、纤维支气管镜、脑CT、骨扫描、腹部超声等检查，除外转移性病变的存在。

### 1.2 治疗方法

采用全身麻醉双腔气管插管，单侧肺通气，患者取健侧卧位。在腋中线第7或第8肋间置戳卡放入胸腔镜，观察有无粘连和病变情况，了解肿瘤的部位、大小、胸膜、心包、肺门结构和纵隔淋巴结是否有肿瘤侵犯或转移。根据病变选择小切口部位，上肺病变选择第4肋间，下肺、中肺病灶选择第5肋间，在腋前线至腋后线之间行6 cm-8 cm小切口，用内镜器械和常规器械相结合进行手术操作，通过胸腔镜器械完成游离下肺韧带、膈面、胸膜顶的粘连等，在直视下进行肺门血管和支气管的解剖以及纵隔淋巴结的清扫，肺叶内淋巴结在游离肺叶过程中将肺叶淋巴结沿血管方向推入待切肺内，随肺叶切除，肺门和纵隔淋巴结清扫在完成肺叶切除后进行。对于肺裂发育好的可用电灼切开，对于肺裂发育不良者，应充分剪开肺门前后胸膜，以肺静脉确定肺裂位置，经肺静脉表面肺实质内做一隧道，用执式切割缝合器或用止血钳夹两侧切开，血管钳夹切断后褥式缝合或用GIA处理，肺血管的结扎或缝扎于胸外打结，用推结器推入胸内，支气管残端用一次性闭合器或GIA处理。标本切除后置于无菌手套内完整取出，以防切口种植转移。术中转移灶或淋巴结送冷冻切片检查，以进行肺癌分期。根据术后病理及淋巴结转移情况，Ⅰa期患者不做特殊治疗，Ⅰb期及以上患者术后6周开始化疗（采用缓解化疗方案^[3, 4]^），4个疗程。

## 2 结果

28例手术均顺利完成，无中转开胸发生，只有1例切口延长至12 cm；所有患者术中及术后均未输血，无心肺功能衰竭、心脑血管意外及感染等严重围手术期并发症发生；手术时间90 min-280 min，平均190 min；淋巴结清扫时间30 min-50 min，平均46 min；术中出血量200 mL-450 mL，平均300 mL；麻醉镇静药使用时间20 h-30 h，平均24 h；胸管留置时间3 d-8 d，平均5 d，术后总引流量300 mL-800 mL，平均460 mL；术后住院7 d-14 d，平均12 d。肿瘤 < 3 cm的有12例，>3 cm的有16例。VAMT手术种类包括:行右肺上叶切除术5例，右肺上中叶切除术1例，右肺中叶切除1例，右肺中下叶切除3例，右肺下叶切除3例，右全肺切除术1例，左肺上叶切除9例，左肺下叶切除5例。共清扫淋巴结582枚，平均21.6枚/例，病理确诊转移的淋巴结为93枚。所有患者术后均得到密切随访，截至2009年5月15日，随访2个月-91个月，平均36个月，死亡10例（均为Ⅱa期及以上患者），其中有2例在术后2个月死亡（1例有肝癌转移，另1例为高龄的Ⅲa期患者），另外8例死者均存活超过1年（12个月-61个月，平均23个月），3年生存率为60.7%（95%CI: 40.3%-81.1%）（图 1）。仍然健在的患者的术后生存期为18个月-91个月，中位生存期为45个月。

**1:**
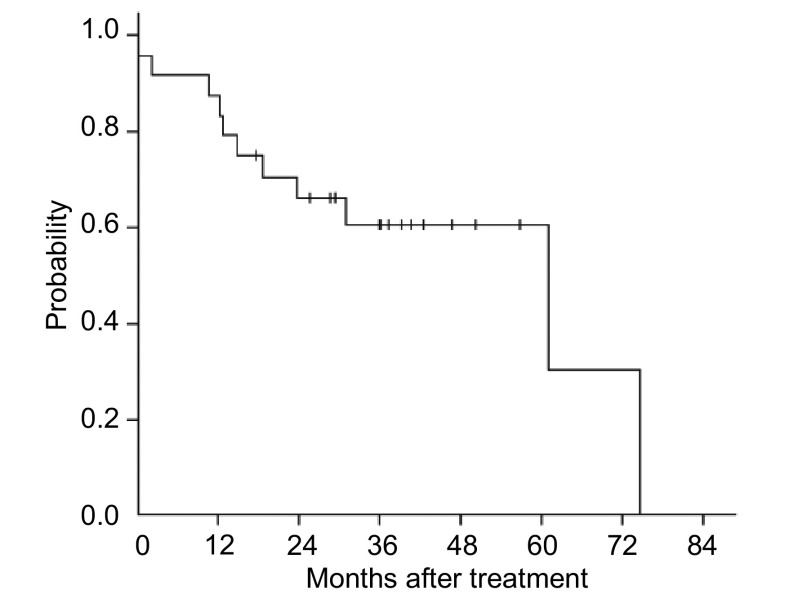
28例多形性肺癌患者生存曲线 Survival curves of 28 patients with pleomorphic carcinoma of the lung

## 3 讨论

1992年Lewis^[5]^首次报道用VATS行肺叶切除术治疗肺癌，因其具有创伤小、恢复快、出血、输血少、对心肺功能影响小、开关胸时间短等优点，所以其治疗效果也比较理想，Ⅰ期肺癌的3年生存率为94%，Ⅱ期为57%，Ⅲ期为25%^[6]^。此后国内外学者也开展了VATS的相关临床研究。历经近20年的不断发展，VATS已成为一门成熟的胸部外科技术和临床常用手术方法之一，被认为是自体外循环问世以来胸外科领域的又一重大技术革新。目前，VATS已被广泛地应用于治疗各种胸部疾病，特别是早期肺癌的治疗^[7]^。不过VATS也具有一定的局限性。有学者^[8]^指出，VATS肺叶切除虽然不会降低患者的生存率，但是会增加淋巴结转移阳性患者局部复发的风险，他们认为可能与VATS操作下淋巴结清扫不如直视操作下更加彻底有关。虽然在VATS下行肺叶切除术和肺门纵隔淋巴结清扫术在技术上是可行的，但是对操作者的技术要求非常高^[9]^，而且，在VATS肺叶切除术中，多数外科医师处理肺血管和支气管时采用的是非解剖性的手术方法，容易造成出血以及不能彻底切除肿瘤。另外还容易造成切口处肿瘤种植。因此，目前大多数单位选择VAMT肺叶切除术。VAMT具有VATS和传统开胸手术的优点，即避免了传统手术的肌肉和神经损伤，创伤小，照明效果好，视野宽且有一定放大作用，可在直视下用常规器械完成肺门及纵隔的操作，缩短手术时间，术后患者恢复快。本组VAMT肺叶切除联合淋巴结清扫治疗28例多形性肺癌患者，无围手术期死亡，无心肺功能衰竭、心脑血管意外及感染等严重围手术期并发症发生，辅以缓解化疗方案，3年生存率高达60.7%。

VAMT在VATS时扩大某一器械操作切口（一般为6.0 cm-8.0 cm），能使常规手术器械和内镜器械相配合使用，通过直视和腔镜联合观察提供清晰视野，精确地判断肿瘤大小、质地、活动度及其与周围的关系，且可用肉眼观察病灶、肺门及纵隔淋巴结，手指探查病灶，区分肿瘤与重要脏器的粘连程度，使探查更接近于常规开胸手术，更好地判断肿瘤的可切除性，降低开胸探查率，它弥补了单纯胸腔镜手术的不足。结合胸壁小切口，在直视下进行胸内操作，一旦手术过程中出血，还可以方便地直视下止血，效果确实可靠。因此VAMT既达到微创的目的，也因为有小切口的辅助，减少操作难度，降低暴露不充分所带来的风险，易于解剖结构的把握，有助于彻底清扫肺门纵隔淋巴结。与常规开胸手术比，VAMT几乎不影响背阔肌、前锯肌、胸大肌等肌群，出血少，术后疼痛轻，利于患者咳嗽排痰和肺功能恢复；骨性胸廓被牵拉的程度也比较小，因此胸廓稳固性好，心肺功能受到的干扰也相对比较小，术后并发症少，恢复快，住院时间缩短^[10, 11]^。可应用常规器械进行胸内操作，对重要血管离断可采用体外简易打结器进行，大大减少了一次性材料及器械的使用，减轻了患者的经济负担。

VAMT扩大了胸腔镜手术的范围，使一些粘连较重，以及过深过大的病变可以通过胸腔镜手术完成。尤其是本组有大部分肿瘤直径>3 cm，非常适合进行VAMT。由于可以直视触摸到病变，对病变的判断及手术方式的决策更加准确。由于其创伤小、恢复快、对高龄、肺功能低及全身情况差及姑息手术患者提供了新的治疗机会。因此，VAMT进行肺癌根治术可使手术难度和风险大大下降。小切口位置选择是顺利实施该手术方式的关键。通过辅助小切口有利于肿块的取出，在肿块取出后可用常规器械进行纵隔暴露与清扫，增加手术彻底性。我们认为手术切口位置首先要有利于术野的暴露及手术操作，尽量在接近病变处做切口，方便使用常规手术器械进行肺叶血管及支气管处理；肺门淋巴结离小切口较近，用常规方法清扫。其次，考虑切口的美学要求及避免切断过多胸壁肌肉，减少手术创伤。切口的长度应根据手术的难易程度及术者对胸腔镜下操作的熟练程度决定。

结合文献及自己的经验，作者认为，VAMT可以完成肺叶切除和全肺切除以及清扫肺门、纵隔淋巴结，对于肿瘤直径较大者，也可达到根治的目的，可以达到与常规开胸手术同样的治疗效果，不过纯VATS要达到同样的效果可能较为困难。但是VAMT仍有一定的局限性，目前尚无法完全代替传统开胸手术。随着胸腔镜手术的广泛开展，各种手术方式和技巧将不断改善和提高，胸腔镜辅助小切口完成标准解剖意义上的肿瘤切除达到常规开胸手术的目的有很好的应用前景。
